# CD161+ regulatory t cells in immune regulation

**DOI:** 10.1186/1546-0096-12-S1-P39

**Published:** 2014-09-17

**Authors:** Chantal Duurland, Tony Brooks, Mike Hubank, Ryan O'Shaughnessy, Lucy Wedderburn

**Affiliations:** 1 Infection, Inflammation and Rheumatology Section, London, UK; 2 UCL Genomics, London, UK; 3 Immunobiology Section, UCL Institute of Child Health, London, UK

## Introduction

Regulatory T cells (Treg) are crucial for maintaining immune homeostasis and mediating immune tolerance. Several studies have shown that Treg can produce cytokines. CD161, a C-type lectin receptor, identifies Treg that produce cytokines. Interestingly, CD161+ Treg are enriched in the inflamed joint of Juvenile Idiopathic Arthritis (JIA) patients and their numbers relate to clinical disease severity suggesting that CD161+ Treg may play an important role in disease pathogenesis. If Treg are to be used as a cell therapy it may be important to define ways to prevent cytokine production from these cells *in vivo*.

## Objectives

To analyse and define the transcriptional and protein signatures of CD161+ Treg compared to CD161- Treg.

## Methods

CD161+ and CD161- Treg were isolated from blood of healthy controls using FACS sorting. Transcriptional differences were analysed using RNA sequence and protein signature by flow cytometry.

## Results

Analysis of transcriptional differences between CD161+ and CD161- Treg highlighted many interesting differentially expressed genes, including Treg-specific genes and genes associated with memory phenotype. RNA and protein expression correlate well for the genes and proteins examined. Pathway analysis of differentially expressed genes indicates the importance of several pathways. For example, epithelial adherence junction signalling, T helper cell differentiation, cell cycle and STAT3 pathway. Future work will focus on exploring the biological importance of these pathways in CD161+ and CD161- Treg in both healthy individuals and JIA patients.

## Conclusion

Analysis of transcriptional differences and protein signature of CD161+ and CD161- Treg cells indicates that these two Treg subpopulations have a different phenotype and may have arisen via different pathways. The generation of a large novel data set concerning transcriptional signatures of CD161+ and CD161- Treg has yielded a rich source of pathways and mechanisms that warrant further investigation and may generate novel targets for drug development.

## Disclosure of interest

None declared.

